# Admission indications, initial diagnoses, Interventions, and patient outcomes within the sole obstetric high-dependency unit in Ethiopia

**DOI:** 10.1186/s12905-024-03175-z

**Published:** 2024-06-06

**Authors:** Zewdu Beza, Roza Tadesse, Henok Teshome, Genetu Tadele, Melkamu Siferih

**Affiliations:** 1 Department of Anesthesiology, Critical Care and Pain Medicine, School of Medicine, Jimma University, Jimma, Ethiopia; 2 Department of Anesthesiology, Critical Care and Pain Medicine, St. Paul’s Hospital Millennium Medical College, Addis Ababa, Ethiopia; 3 Hepatobiliary Surgery, Department of General Surgery, St.Paul’s Hospital Millennium Medical College, Addis Ababa, Ethiopia; 4 Department of Obstetrics and Gynecology, School of Medicine, Jimma University, Jimma, Ethiopia; 5 Department of Obstetrics and Gynecology, School of Medicine, Debre Markos University, Debre Markos, Amhara, Ethiopia

**Keywords:** Obstetric high-dependency unit, Ethiopia

## Abstract

**Background:**

Obstetric high-dependency care offers holistic care to critically ill obstetric patients while maintaining the potential for early mother-child bonding. Little is known about the obstetric high-dependency unit (HDU) in Ethiopia. Therefore, the objective of the study was to review the admission indications, initial diagnoses, interventions, and patient outcomes in the obstetric high-dependency unit at St.Paul’s Hospital.

**Methods:**

A retrospective observational study was carried out at St. Paul’s Hospital in Addis Ababa, Ethiopia, between September 2021 and September 2022, targeting patients in the obstetric high-dependency unit during pregnancy or with in 42 days of termination or delivery. A checklist was used to compile sociodemographic and clinical data. Epidata-4.2 for data entry and SPSS-26 for data analysis were employed. Chi-square tests yielded significant results at *p* < 0.05.

**Result:**

Records of 370 obstetric patients were reviewed and analyzed. The study enlisted participants aged 18 to 40, with a mean age of 27.6 ± 5.9. The obstetric high-dependency unit received 3.5% (95% CI, 3.01-4.30) of all obstetric admissions. With the HDU in place, only 0.42% of obstetric patients necessitated adult intensive care unit (ICU) admission. The predominant motive behind HDU admissions (63.2%) was purely for observation. Hypertensive disorders of pregnancy (48.6%) and obstetric hemorrhage (18.9%) were the two top admission diagnoses. Ten pregnant mothers (2.7%) were admitted to HDU: 2 with antepartum hemorrhages, and 8 with cardiac diseases. Maternal mortality and transfer to the ICU were both 1.4 per 100 HDU patients.

**Conclusion:**

Our study found that the most frequent indication for admission to the HDU was just for observational monitoring. Hypertensive disorders of pregnancy and obstetric hemorrhage were the two leading admission diagnoses. Expanding HDUs nationwide is key for mitigating the ICU burden from obstetric admissions. Strategies for early prenatal screening, predicting preeclampsia, and addressing postpartum hemorrhage should be reinforced. Future studies should focus on a broader array of factors affecting fetomaternal outcomes in such a unit.

## Introduction

Due to a rise in complicated pregnancies and resulting maternal mortality, there is a heightened demand for admissions to maternal critical care units, leading to increased healthcare expenditure [1, 2]. Between 1 and 9 out of every 1000 deliveries necessitate admission to intensive care units (ICUs), with obstetric complications constituting the majority of admission diagnoses [3]. Whereas intensive care units (ICUs) are available in the majority of tertiary hospitals, many critically ill obstetric patients must wait for ICU beds due to a lack of ICU beds and personnel [2, 4–9].

Obstetric intermediate care units, frequently referred to as obstetric high-dependency units (HDU), were developed to achieve a greater equilibrium between patient outcomes, client needs, and logistical or financial limitations. Patients with a single organ failure and those who are at a high risk of developing life-threatening complications are typically included [5–15]. Patients requiring high-dependency unit (HDU) placement have conditions that surpass the capabilities of general wards but don’t necessitate the level of care provided in ICUs, which is primarily geared toward addressing organ failure. These units are limited, particularly in developing countries [12, 13, 16].

The staffing requirements for an obstetric ICU unit involve one anesthesiologist or intensivist, one nurse per bed per shift, and other supporting staff members. Conversely, in the HDU, one nurse is designated for every two beds per shift, alongside other staff members, with no requirement for an anesthesiologist or intensivist [12].

There are many conceivable rewards to having an HDU in an obstetric context, including the simultaneous provision of skilled obstetric care and critical care. Patients would not be required to be taken to a conventional medical/surgical ICU as often, enabling on-site monitoring and quick, logical care for patients with hemodynamic instability [17, 18]. This unit is handled by anesthesia and obstetric consultants and is close to the operating rooms and the postoperative recovery area [13]. There is a reduction in ICU admission likely related to the corresponding increase in maternal admission to HDU [16]. It also offers obvious benefits of keeping mother and child together and ensuring greater continuity of prenatal and postnatal care [13, 14].

Ethiopia has one of the highest maternal mortality ratios in sub-Saharan Africa, notwithstanding recent advances in maternal mortality reduction (267 deaths per 100,000 live births, according to the most recent United Nations estimate for 2020) [19]. The country is dedicated to achieving the Sustainable Development Goals on Maternal Mortality Ratio (MMR: 70 per 100,000 live births by 2030) [20, 21]. Many in-depth studies of maternal deaths have called for the establishment of dedicated on-site obstetric care to mitigate maternal morbidity and mortality [1, 2, 14, 16]. In Ethiopia, the majority of general ICUs cannot fully accommodate level 2 obstetric patients, and there is a notable lack of ICUs exclusively dedicated to obstetric care. However, there is just one obstetric HDU in the entire country, which is found in Addis Ababa at St.Paul’s Hospital Millennium Medical College. Within our context, there was a notable absence of data regarding how the obstetric HDU could play a pivotal role in reducing severe maternal morbidity and mortality.

Evaluating the characteristics and outcomes of critical obstetric patients admitted to high-dependency units within low-income contexts holds promise for discerning priorities and resource requirements essential for advancing healthcare provision. Obstetric critical care data offers invaluable guidance for crafting effective policies [11].

Thus, the current study aimed to review admission indications, initial diagnoses, interventions, and patient outcomes in the obstetric HDU in St.Paul’s Hospital Millennium Medical College.

## Methods and materials

### Study setting, design, and participants

A retrospective observational study was conducted at St.Paul’s Hospital Millennium Medical College, Addis Ababa, the capital city of Ethiopia, between September 15, 2021, and September 15, 2022. It is one of the biggest tertiary hospitals in the country. There are several complicated patients in the obstetric service at this hospital. The hospital took proactive measures in September 2021 by inaugurating an obstetric high-dependency unit to meet the increasing demand for standard of care, address the nation’s high maternal mortality rate, and relieve pressure on ICUs handling obstetric cases. This critical care unit is located in the obstetric department, near the operating rooms and the postoperative recovery area. It has a total of four beds equipped with two mechanical ventilators; whereas the normal adult ICU has a 15-bed unit run by critical care physicians. The HDU was staffed by one Maternal-Fetal Medicine expert, one anesthesiology resident, and four nurses. Throughout the study period, pregnant or postpartum individuals admitted to the obstetric high-dependency unit, including those undergoing pregnancy, delivery or termination within 42 days, were eligible for participation. The exclusion criterion hinged on the completeness of patient records. It was essential to include admission diagnoses, management strategies, and patient outcomes as relevant variables. During the study period, 370 obstetric patients were admitted to the obstetric HDU. Every patient was included consecutively since they all met the inclusion criteria. The necessary data was taken from the hospital records (case notes and records from the intensive care unit and the high-dependency unit.

### Study variables

The dependent variable was the outcome for critically-ill obstetric patients in the HDU, categorized as discharged improved, died after admission to the HDU, or trannsferred to the ICU. Age, parity, residence, admission indication, admission diagnosis, mode of delivery or handling the index pregnancy, gestational age at delivery or termination, type of intervention, length of stay in the HDU, and the immediate cause of death were all considered independent variables.

### Data collection tool and data quality assurance

A structured checklist was devised after reviewing the pertinent literature [2, 14, 16, 18, 22–25]. The checklist was prepared in the English language and contained sociodemographic data and clinical profiles such as admission indications, diagnoses at admission, intervention modalities, length of stay, and patient outcomes in terms of discharged improved, died after HDU admission or transferred to the general ICU. Two certified nurses and one anesthesiology resident were recruited for data collection and supervision after training and proper orientation. The training aimed to familiarize them with research objectives and the process of data collection. Upon completing the data collection process, daily reviews were conducted to confirm the completeness and consistency of each checklist and the general data quality.

### Operational definition

A mechanically ventilated patient with respiratory failure or who has had two or more organ failures is cared for in an intensive care unit (ICU) by one intensivist or anesthesiologist and one nurse. High-dependency unit is a setting where critically ill patients are treated for organ failures other than respiratory failure with the help of one nurse and two beds, without the need for intensivists or anesthesiologists. Maternal death (obstetric death) is death that occurs in a woman from any cause connected to or aggravated by the pregnancy or its management (excluding accidental or incidental causes) during pregnancy and childbirth or within 42 days of termination of pregnancy, regardless of the duration and site of the pregnancy [26]. Patient care at Level 2 includes care that is tailored to provide support for a single organ [12].

### Data processing and statistical analysis

Data were double-entered and cleaned, with Epidata version 4.2, and exported to SPSS version 26 for analysis. With descriptive statistics like frequency, percentage, mean, median, and interquartile range, the data were summarized, tabulated, analyzed, and expressed. Frequencies and percentages for categorical variables, the mean for continuous variables with normally distributed data, the median, and the interquartile range for data with a non-normal distribution were all presented. Pearson Chi-square tests were used in the bivariate analysis to compare the outcomes of obstetric patients with their admission indications and admission diagnoses. Statistical significance was defined as a p-value of 0.05 or less.

## Results

### Socio-demographic and clinical characteristics

A total of 370 obstetric patient records were meticulously reviewed and included in the final analysis, attaining a documentation rate of 100%. Postnatal and postoperative admissions accounted for 97.3% of study participants. The review encompassed 10,442 admissions for obstetrical care. Over the study period, admissions to the HDU accounted for 3.5% of all obstetric admissions (95% CI, 3.01–4.30). Within the timeframe of 2021 to 2022, adult ICU admissions, which encompassed 44 obstetric cases, represented only 0.42% of the total obstetric admissions.

The study participants ranged in age from 18 to 40, with a mean age of 27.6 ± 5.9. 63% of patients (63.5%) fell between the 20- to 35-year-old age range. The majority (71.6%) of patients lived in an urban area. Patients who were multiparous comprised more than half of the participants (58.9%). Cesarean sections were used to deliver the majority of patients (66.6%). Ectopic pregnancies accounted for 1.1% of admissions to the HDU obstetrics unit, each requiring exploratory laparotomy. Among abortion cases, 5 patients underwent hysterotomies, 4 underwent dilation and evacuation, and three underwent MVA. Over half of patients (57.3%) had gestational ages between 28 and 34 weeks at delivery. The median time spent in the HDU was 36 h (IQR: 24–60). Nearly half (44.6%) of the patients stayed at the HDU for a maximum of 24 h. About 7% of patients lasted for more than 7 days (Table 1).

**Table 1:** Sociodemographic and clinical characteristics of obstetric patients in the high-dependency unit (*n* = 370)

Variables		Frequency	Percentage (%)
Age	≤ 19	111	30
	20–35	235	63.5
	> 35	23	6.2
Parity	Nulliparous	7	1.9
	Primiparous	146	39.5
	Multiparous	218	58.9
Address	Urban	265	71.6
	Rural	105	28.4
Mode of delivery or handling of the index pregnancy	Cesarean section	246	66.5
	Vaginal delivery	108	29.2
	Exploratory laparotomy for ectopic pregnancy	4	1.1
	Abortion care (MVA, D&E or hysterotomy)	12	3.2
Gestational age at delivery	< 28	16	4.3
	28–34	213	57.6
	34–36 wks + 6days	58	15.7
	≥ 37	90	24.3
Duration of stay in the HDU	< 24 h	165	44.6
	24–48 h	107	28.9
	> 48 h	97	26.2

## Admission indications and initial diagnoses

The majority of patients (97.3%) were admitted to the hospital on an emergency basis. Four individuals (1.1% of the total) necessitated readmission to the obstetric HDU after their discharge to home or transfer to a ward. Within the readmitted cases, there were two instances of disseminated intravascular coagulation (DIC), two cases of sepsis, and one occurrence of eclampsia. A choice was made to extract just one sample showcasing the more serious indication or complication from the documentation.

The most frequent reason for admission to HDU was just for observation (63.2%). Yet, nearly a third (27.8%) of individuals suffering from serious organ dysfunction depended on assistance for breathing. Obstetric-related conditions accounted for the majority of admission diagnoses (77.8%). At the time of admission, diagnoses regarding hypertensive disorders of pregnancy made up 48.6% of the whole. Preeclampsia was the diagnosis for 27% of those who were hospitalized to the HDU. Obstetric hemorrhage was indicated as the primary diagnosis in 18.9% of admissions to the HDU, of which post-partum hemorrhage accounted for 93.5%, antepartum hemorrhage for 2.9%, and ectopic pregnancy for 3.6%. Cardiac disease affected around 16.8% of the individuals. Patients with HELLP syndrome made up 13.5% of the participants, while those with eclampsia constituted 8.1%. Ten pregnant mothers were among the patients admitted to the HDU, constituting 2.7% of all admissions. Of these, two experienced antepartum hemorrhage (both placenta previa), while eight presented with cardiac conditions (Table 2).

**Table 2:** Admission indications and initial diagnoses (*n* = 370)

Admission indications	Frequency	Percentage (%)
Respiratory support	103	27.8
Cardiovascular support	84	22.6
Observation only	234	63.2
Airway protection	59	15.9
Initial diagnoses		
Preeclampsia	100	27
Obstetric hemorrhage	70	18.9
Cardiac disease	62	16.8
HELLP syndrome	50	13.5
Chorioamnionitis	10	2.7
Sepsis	15	4.1
Respiratory disease	20	5.4
Acute kidney injury	7	1.9
Disseminated intravascular coagulation	2	0.5
Eclampsia	30	8.1
Uterine perforation	2	0.5
Uterine rupture	3	0.8

### Interventions

Antibiotics were administered to the vast majority of patients (83.8%) who stayed in the HDU. MgSO4 was provided to 48.6% of participants. For 36.8% of patients, blood transfusions were necessary. 72% of patients did not require oxygen support. Mechanical ventilation was necessary for 6.3% of admitted patients (Table 3).

**Table 3:** Intervention types (*n* = 370)

Specific intervention	Frequency	Percentage (%)
Blood transfusion	136	36.8
Fluid resuscitation	10	2.7
Vasopressor support	7	1.9
Oxygen support	103	27.8
Mechanical ventilation	23	6.2
Face mask/nasal oxygen	80	21.6
Antibiotics	310	83.8
MgSO4	180	48.6
Hemodialysis	10	2.7

### Patient outcomes in the obstetric high-dependency unit

For every 100 HDU admissions, 1.4 patients were transferred to the general ICU, and the crude maternal mortality rate was 1.4 per 100 admissions. All maternal deaths were after being transferred to the conventional ICU. Significant associations between the survival, the type of intervention employed, and the admission indications were found. The HELLP syndrome was present in virtually every deceased obstetric patient. In addition, sepsis was found in four of the five deaths, eclampsia in three of them, and obstetric hemorrhage in two of them. The immediate causes of death in the groups of patients with sepsis and hypertensive disorders during pregnancy were cardiovascular collapse followed by multiple organ failure in two cases and cerebral hemorrhage in one case. In the obstetric hemorrhage group, the two causes of death were hypovolemic shock and renal failure (Table 4).

**Table 4:** Correlation between the admission indication, the intervention strategies, and the patient outcomes (Pearson Chi-square tests)

Admission indications	Discharged improved	Died	*P*-value
Respiratory support	100(97.1)	3(2.9)	0.044 0.01
Cardiovascular support	84(94.4)	5(5.6)	
Observation only	233(99.6)	1(1.1)	
Airway protection	55(93.2)	4(6.8)	
Intervention strategies	Discharged improved	Died	
Blood transfusion	132(97.1)	4(2.9)	
Fluid resuscitation	7(8.8)	3(1.2)	
Vasopressor support	4(57.1)	3(42.9)	
Oxygen support	103(97.2)	3(2.8)	
Antibiotics	305(98.4)	5(1.6)	
MgSO4	290(98.6)	4(1.4)	
Hemodialysis	5(0.5)	5(0.5)	

Numbers in the parenthesis are percentages

## Discussion

This study was designed to evaluate and pioneer exploration into the admission indications, initial diagnoses, care approaches, and patient outcomes in the lone obstetric high-dependency unit situated at St.Paul’s Hospital Millennium Medical College in Ethiopia. The nation is embarking on the initial phases of obstetric HDU development. An obstetric high-dependency unit serves to strengthen the maternal-infant bond while also ensuring a seamless continuum of care from prenatal to postnatal stages, representing two key advantages. The accessibility of HDUs significantly contributes to bolstering the chances of survival for obstetric patients facing critical conditions in regions such as Ethiopia, characterized by constrained healthcare resources and prevalent serious illness.

Despite the acknowledged advantages of HDU, our study revealed that the primary reason for admission for the majority of patients was for observational monitoring. Hypertensive pregnancy disorders, notably preeclampsia, and obstetric hemorrhage, particularly postpartum hemorrhage, stood out as the prevailing diagnoses at admission. The leading non-obstetric condition identified was cardiac disease. Mortality predominantly resulted from obstetric causes. For HDU admissions, both the transfer rate to the general ICU and the crude maternal mortality rate were 1.4 per 100 admissions.

Across the previous five years (2016–2020), adult ICU admissions constituted 0.94% of the total obstetric admissions. The introduction of the HDU led to a noticeable reduction in adult ICU admissions, with numbers dropping by 55.3% compared to the average of the past five years (95% CI, 48.9–58.3, *p* = 0.01), and a subsequent reduction of 38.2% observed in 2021 (95% CI, 32.4–43.6, *p* = 0.03). The findings imply the importance of emphasizing the utilization of obstetric high-dependency units (HDUs) and underscore the necessity for nationwide expansion to relieve the pressure on ICU admissions for obstetric patients.

Our study found that 3.5% (95% CI, 3.01–4.30) of obstetric patients were admitted to the HDU, aligning closely with the 4.2% reported in the UK Survey [18], despite pronounced socioeconomic discrepancies. Nevertheless, it demonstrated a notable difference from the two studies conducted in India (11.1% and 11.2%) [6, 27], as well as those in the UK (5.01%) [7], and China (7.1%) [14]. Factors such as differences in capacity among obstetric HDUs, demographic diversity within populations, varying admission or transfer criteria for HDUs or ICUs, fluctuations in the establishment and quantity of obstetric HDUs in different regions, the distinct catchment areas designated for each obstetric HDU, and variations in annual birth rates can account for the observed discrepancies.

Compared to the other studies (which varied from 10 to 20%) [7, 13, 28], our obstetric HDU demonstrated a remarkably low antenatal admission rate of 2.7%. One potential explanation for this could be the effectiveness of our antenatal care program. Such particulars might be elucidated within a unique follow-up protocol needed for antenatal patients during labor, continuous fetal monitoring, ensuring the presence of essential equipment in the delivery ward, a dedicated obstetric team, and the requirement for cardiotocography. Another inference is that our obstetric HDU should expand its staffing diversity and incorporate rigorous fetal monitoring to accommodate a broader spectrum of prenatal patients in the HDU. Conversely, enhancements in prenatal screening and diagnostics within the set-up might detect issues at an earlier stage, enabling timely intervention and preventing severe complications that might otherwise lead to hospitalization.

Consistent with the previous studies [5, 11, 14, 23, 29], our study found that the principal causes of HDU admissions were obstetric factors, notably hypertensive disorders during pregnancy, predominantly preeclampsia, followed by obstetric hemorrhage, particularly postpartum hemorrhage. Likewise, HELLP syndrome and eclampsia made substantial contributions to hypertensive disorders in pregnant women. However, this is in contrast to a study from Eastern India, where sepsis was the main diagnosis at admission (35.08%), followed by postpartum hemorrhage (29.82%) and hypertensive disorders of pregnancy (21.05%) [6]. The variation may be linked to the socioeconomic conditions and diagnostic strategies employed within our context, where clinical suspicion is the primary mode of diagnosis. Moreover, in our study, a predominant number of participants lived in urban areas, where the likelihood of encountering sepsis risk factors such as malnutrition and anemia appears to be lower, and where most births are attended to healthcare facilities equipped with a relatively better infrastructure. However, data from a study at the University of Birmingham Women’s Hospital in the UK showed that obstetric hemorrhage was the leading diagnosis prompting hospital admissions [7]. This finding contrasted with ours, potentially because of differences in risk factors across studies, including differing rates of cesarean deliveries, occurrences of placenta previa, multiparty, pregnancies facilitated by assisted reproductive techniques, individuals with complex medical conditions during pregnancy, and the efficacy of the HDU in tracking hemodynamic instability. Cardiac disease emerged as the primary cause of non-obstetric complications leading to HDU admissions, reflecting patterns noted in East India and the UK [6, 7].

A substantial proportion of patients were observed at the HDU for a maximum of 24 h. This differs from earlier research findings [8, 15, 30], where the typical length of hospitalization for the majority of patients fell within the range of 4 to 7 days. The bulk of our facility’s patients were admitted primarily for observation purposes, requiring shorter stays. Furthermore, it is important to highlight that 7% of patients warranted monitoring for a duration exceeding 7 days. A plausible explanation is that our patients experienced severe complications, including HELLP syndrome, eclampsia, sepsis, and acute kidney injury necessitating hemodialysis, alongside various other challenges.

Transfer to the general ICU was necessary in 1.4 out of every 100 admissions, which is substantially higher than in the UK study (1.4 per 1000 admissions) [7]. The rationale behind this could be that, instead of calculating the average rate over multiple years, our study tracked the transfer rate specifically within a single year. An alternative reason could be the differences in the severity of individual obstetric complications, which require invasive monitoring, alongside variations in annual birth rates.

The mortality after HDU admissions in our study exceeded that reported in two separate studies from China [14, 31], and Ireland [13]. The key explanation likely revolves around admitting twenty-three mechanically ventilated patients to our obstetric high-dependency unit as a measure to alleviate the extensive burden on the adult ICU. Another possible distinction could be linked to the overall socioeconomic development and the extent to which HDUs are utilized. Obstetric factors accounted for all maternal deaths observed in our study. The current finding challenges the conclusions drawn in the preceding Chinese study [14], which identified cardiac diseases as the predominant cause of death. The inconsistency may be explained by the predominance of admission diagnoses or indications related to obstetric reasons in our study. Alternatively, it is conceivable that women with non-obstetric conditions in prior research exhibited a higher tendency of complications compared to their counterparts with obstetric causes. However, it bore resemblance to studies conducted in Kenya [15], in Costal India [32], and another study in India [33], indicating that HELLP syndrome and obstetric hemorrhage emerged as major culprits in maternal mortality.

One of the study’s strengths is its novelty in providing data from a tertiary care environment in Ethiopia, notably with the implementation of obstetric HDU. The primary shortfall of our study was its retrospective nature and exclusive focus on a single center. In addition, no standardized criteria were in place for admitting patients to the high-dependency unit. It was no longer capable of assessing newborn outcomes, as well as the knowledge, attitudes, and perceptions of healthcare providers, along with cause-specific reductions in ICU admissions. Because there was a scarcity of similar studies conducted locally, it proved difficult to draw comparisons. Given these limitations, it is crucial to approach the findings of this study with caution.

## Conclusion

Our study reviewed admission indications, initial diagnoses, interventions, and patient outcomes within the sole obstetric HDU in Ethiopia. The most frequent indication for admission to HDU was just for observation. Hypertensive disorders of pregnancy and obstetric hemorrhage were the two leading admission diagnoses. Nationwide expansion of HDUs is essential as it helps reduce the burden on ICU admissions for obstetric patients. The strategies for early antepartum screening, preeclampsia prediction, and postpartum hemorrhage prevention should be reinforced. Future research endeavors should emphasize conducting multicenter studies that take into account a broader array of factors influencing fetomaternal outcomes, alongside exploring healthcare providers’ knowledge and perceptions and delving into the cause-specific role of HDU.

## Data Availability

Data can be made available from the corresponding author on reasonable request.

## Abbreviations

DIC: Disseminated Intravascular Coagulation
HDU: High Dependency Unit
HELLP: Hemolysis, Elevated Liver Enzymes, Low platelets
ICU: Intensive Care Unit
IQR: Interquartile Range
MgSO4: Magnesium Sulphate
MMR: Maternal Mortality Ratio
MVA: Manual Vacuum Aspiration
SPSS: Statistical Package for Social Sciences
